# The Responsibility to Transform: Ethics and Training in Plastic Surgery

**DOI:** 10.7759/cureus.101098

**Published:** 2026-01-08

**Authors:** Celina V Kishi

**Affiliations:** 1 Education and Clinical Research Department, Instituto Jalisciense De Cirugía Reconstructiva, Guadalajara, MEX

**Keywords:** aesthetic norms, cultural influences, decision-making, ethical training, hidden curriculum, narrative competence, plastic surgery ethics, professional identity, surgical judgment, training environment

## Abstract

Plastic surgery occupies a distinctive ethical space in medicine, where interventions alter appearance, identity, and social meaning. Yet surgical training has traditionally emphasized technical skill while leaving the cultural and moral dimensions of transformation insufficiently explored. This narrative review examines the ethical foundations of aesthetic and reconstructive practice and the pressures that shape surgical judgment, including the hidden curriculum, globalized beauty ideals, and gendered dynamics in training environments. These forces narrow patient autonomy and normalize aesthetic templates that may obscure individual identity. We propose a concise framework for ethical training that links technical safety with reflective practice and positions the patient’s narrative at the center of decision-making. Ethical formation strengthens, rather than competes with, surgical excellence by fostering discernment, cultural awareness, and respect for individuality. The responsibility to transform applies not only to patients but to the surgeons whose professional identity is shaped through training.

## Introduction and background

Plastic surgery occupies a unique position within medicine as a field in which surgical interventions modify not only anatomy but also identity, social meaning, and personal agency [1]. Although traditional training has emphasized technical proficiency and procedural safety, growing evidence indicates that ethical reasoning, cultural interpretation, and narrative understanding are equally essential to responsible clinical practice and patient-centered decision-making [2].

Contemporary aesthetic and reconstructive decisions are shaped by globalized beauty norms, institutional hierarchies, digital image culture, and the hidden curriculum, defined as the implicit transmission of values, norms, and expectations within training environments through role modeling, hierarchy, and informal practices [3,4]. These influences affect how surgeons interpret patient requests, define acceptable indications, and evaluate aesthetic outcomes. When unexamined, they may constrain clinical judgment, promote standardized aesthetic templates, and limit the ability to recognize patients as individuals with diverse motivations, identities, and psychosocial contexts.

Recent scholarship in surgical education has identified important gaps in ethical preparedness among residents, including limited exposure to reflective practice, insufficient training in patient-centered judgment, and inadequate recognition of how cultural narratives influence expectations, consent, and postoperative satisfaction [5]. However, despite these insights, existing literature often remains fragmented. Perspectives from surgical ethics, medical humanities, sociology of the body, and cognitive psychology are rarely integrated into a unified analysis of surgical judgment, limiting the translation of ethical theory into clinical practice.

Therefore, the objective of this narrative review is to integrate current interdisciplinary evidence from surgical ethics, cultural studies, cognitive science, and professional identity formation to examine how ethical reasoning develops in plastic surgery training and practice. Specifically, we analyze how external cultural pressures, the hidden curriculum, and cognitive biases influence clinical decision-making; assess the strengths and limitations of existing research; and identify gaps that must be addressed to inform more comprehensive and ethically grounded surgical training frameworks. This approach emphasizes that technical competence alone is insufficient and that ethical formation is fundamental to patient safety, professional identity, and responsible aesthetic and reconstructive practice.

## Review

Ethical judgment in aesthetic and reconstructive decision-making

Ethical judgment in plastic surgery extends beyond technical proficiency and requires the ability to interpret patient motivations, contextualize clinical risk, and negotiate expectations shaped by social and cultural pressures. Recent literature demonstrates that digital media, social networks, and algorithm-driven beauty filters significantly distort self-perception and contribute to increased demand for standardized cosmetic outcomes [6,7]. These findings highlight a central ethical tension between respecting patient autonomy and recognizing the influence of culturally induced desires.

However, most available studies lack longitudinal designs capable of correlating preoperative motivations with long-term psychosocial outcomes or patient-reported satisfaction. Existing analyses addressing surgical judgment emphasize the ethical responsibility of declining procedures that may compromise patient well-being, yet many rely primarily on expert opinion rather than empirical evaluation [4]. A persistent limitation across this body of literature is the absence of structured, evidence-based frameworks to guide surgeons through ethically complex consultations in a consistent and reproducible manner.

Cultural norms, aesthetic ideals, and their influence on clinical reasoning

Sociocultural scholarship provides essential context for understanding how aesthetic ideals are constructed, disseminated, and normalized. Historical analyses of the global beauty industry describe the standardization and commercialization of beauty across cultures [8], while feminist and sociological perspectives demonstrate how aesthetic labor is gendered and embedded within broader power structures [9,10]. Psychological research further shows that repeated exposure to idealized imagery, including so-called “fitspiration,” negatively affects body image and reinforces internalization of unrealistic standards [11,12].

Collectively, these processes operate through mechanisms of symbolic power, social reproduction, and gendered hierarchies that shape aesthetic norms and expectations [13]. Although these theoretical frameworks are robust, they are rarely incorporated into surgical education or clinical training. As a result, forms of symbolic violence may persist unexamined within professional environments, subtly reinforcing normative aesthetic templates [14]. Despite their clear relevance, these insights are infrequently applied to patient counseling or operative decision-making in plastic surgery, particularly regarding the internalization of beauty ideals and objectification processes [15]. This disconnect limits surgeons’ capacity to critically evaluate the cultural assumptions that inform both patient requests and professional norms.

Professional identity formation and the hidden curriculum in surgical training

Professional identity in surgery is shaped not only through formal curricula but also through the hidden curriculum, defined as the implicit transmission of norms, behaviors, and values within training environments [5,8]. Studies in surgical education consistently demonstrate that gender bias and microaggressions persist despite formal equity initiatives, influencing access to mentorship, operative autonomy, and leadership opportunities [6,16].

Research on professional identity formation further indicates that residents often internalize ethical attitudes and decision-making norms modeled by senior surgeons without explicit discussion or structured reflection [5]. While these studies provide valuable insight into the social dynamics of surgical training, they are frequently limited by qualitative designs and single-institution samples, which restrict generalizability. Notably, there remains a marked paucity of data from Latin American training programs, where cultural, institutional, and social contexts differ substantially from those in North America and Europe, representing an important gap in the literature.

Cognitive biases and limitations in surgical decision-making

Evidence from cognitive psychology and medical education demonstrates that surgeons, like all clinicians, are vulnerable to cognitive biases such as anchoring, confirmation bias, and overconfidence [17,18]. These biases can negatively influence diagnostic accuracy, operative planning, and patient selection. Early-stage trainees, in particular, may exhibit inflated confidence in their aesthetic judgment, which has been associated with suboptimal clinical decision-making [19].

Although these vulnerabilities are well-described, few surgical training programs explicitly address cognitive bias or incorporate reflective strategies designed to mitigate its impact. Existing studies are analytically rigorous but largely descriptive, with limited evidence evaluating educational interventions that improve ethical judgment or reduce bias in real-world clinical settings.

Synthesis of themes and identification of gaps

Across the reviewed literature, several consistent themes emerge. External cultural pressures exert a significant influence on patient motivations and prevailing aesthetic norms. The hidden curriculum plays a decisive yet insufficiently examined role in shaping surgeons’ ethical reasoning and professional identity. Cognitive biases remain underaddressed within surgical education despite clear implications for patient safety and decision quality. Finally, there is a notable absence of integrative frameworks linking ethics, culture, psychology, and surgical training.

Substantial gaps persist, including limited empirical research connecting cultural theory to measurable surgical outcomes, scarcity of studies evaluating structured ethical training interventions, minimal representation of low- and middle-income countries, particularly in Latin America, and a lack of longitudinal research examining how ethical reasoning evolves throughout surgical training. Collectively, these findings reinforce that technical competence alone is insufficient for ethical practice. Ethical formation must be addressed explicitly through structured reflection, narrative-based learning, case-based ethical analysis, and sustained engagement with the cultural forces that shape aesthetic norms.

## Conclusions

Plastic surgery training must extend beyond technical competence to incorporate ethical formation, cultural awareness, and reflective judgment as core components of professional development. Surgeons operate at an intersection where bodily transformation carries profound personal and social meaning, and clinical decisions may either reinforce standardized aesthetic norms or respect individual identity and autonomy. The evidence reviewed demonstrates that cultural pressures, the hidden curriculum, and cognitive biases shape surgical reasoning in ways that remain insufficiently addressed within formal training structures.

Integrating structured ethical reflection, narrative-based learning, and critical engagement with sociocultural influences can strengthen clinical judgment and patient-centered care. Ethical formation should not be viewed as separate from technical excellence, but as integral to safe, responsible, and humane surgical practice. As aesthetic and reconstructive surgery continues to evolve within increasingly complex social contexts, the responsibility to transform extends beyond operative skill to the deliberate cultivation of surgeons capable of ethical discernment, cultural awareness, and reflective, patient-centered decision-making.
